# The protective effect of VSL#3 on intestinal permeability in a rat model of alcoholic intestinal injury

**DOI:** 10.1186/1471-230X-13-151

**Published:** 2013-10-20

**Authors:** Bing Chang, Lixuan Sang, Ying wang, Jing Tong, Dai Zhang, Bingyuan Wang

**Affiliations:** 1 Department of Gastroenterology, First Affiliated Hospital of China Medical University, 110001 Shenyang, Liaoning Province, China; 2 Department of Cadre Ward II, First Affiliated Hospital of China Medical University, 110001 Shenyang, Liaoning Province, China

**Keywords:** Intestinal barrier, TNFα, Tight junctions, VSL#3, Glutamine

## Abstract

**Background:**

This study aimed to investigate the mechanism of the probiotic VSL#3 in acute alcoholic intestinal injury, and evaluate the effect of VSL#3, glutamine,VSL#3+glutamine and heat-killed VSL#3 therapy in a rat model.

**Methods:**

Six- to eight-week-old male wild-type rats were divided into seven groups. To establish the acute alcohol liver disease model, rats received three doses of corn starch dissolved in PBS/40% alcohol administered intra-gastrically every 12 hours. Treatment groups received an intra-gastric dose of VSL#3, Glutamine, heat-killed VSL#3, or VSL#3+Glutamine 30 minutes prior to alcohol administration. The placebo group was treated with PBS prior to alcohol administration. TNFα and endotoxin in plasma was measured by ELISA and Tachypleus Ameboctye Lysate assays, and electron microscopy, Western blotting, and reverse transcription polymerase chain reaction were used to identify the mechanisms of VSL#3 in the regulation of epithelial permeability.

**Results:**

First, compared with control group, endotoxin and TNFα in alcohol group was obviously high. At the same time, in VSL#3 group,the expression of endotoxin and TNFα obviously lower than the alcohol group. And the trends of the expression of tight junction proteins in these groups were reversed with the change of endotoxin and TNFα. Second, compared the groups of VSL#3 with glutamine,VSL#3+glutamine and heat-killed VSL#3,we found that both VSL#3 and heat-killed VSL#3, glutamine were as effective as VSL#3+glutamine in the treatment of acute alcohol liver disease, the expression of endotoxin and TNFα were lower than the alcohol group, and tight junction proteins were higher than the alcohol group whereas the expression of tight junction proteins were higher in VSL#3 + glutamine group than either agent alone, but have no significant difference.

**Conclusion:**

We conclude that VSL#3 treatment can regulate the ecological balance of the gut microflora, preventing passage of endotoxin and other bacterial products from the gut lumen into the portal circulation and down-regulating the expression of TNFα, which could otherwise down-regulate the expression of tight junction proteins and increase epithelial permeability.

## Background

Alcohol consumption is associated with the development of various medical disorders including alcoholic liver disease (ALD) and pancreatitis. Several studies have shown that the phenomenon of short-term excessive drinking is more common than addiction to alcohol, and that there is not always a dose-effect relationship between liver injury and alcohol, environmental, and genetic factors [1,2]. There are currently no effective treatments for these diseases related with alcohol and, other than abstention, no preventive measures. The study presented here describes a potential new target for preventing alcoholic intestinal injury.

There is increasing evidence that the intestinal barrier plays a central role in the initiation of alcohol-induced tissue damage, and this role is most convincing for liver injury. Disruption of the intestinal barrier allows endotoxin and other bacterial products in the gut lumen to pass into the portal circulation and cause hepatic inflammation and the development of alcoholic steatohepatitis (ASH). This in turn can lead to alcoholic cirrhosis and liver failure, which is a causal factor in the development of alcoholic endotoxemia and hepatitis. Keshavarizian [3] found that intestinal barrier dysfunction was observed in alcoholics who also had liver disease. The intestinal barrier is formed by the epithelial cells and the tight junctions (TJs) between them [4], and provides barrier functions between luminal triggers and the host. Intestinal barrier dysfunction may lead to increased uptake of luminal antigens that promote mucosal inflammation.

The human gut harbors a large and dynamic bacterial community which plays a major role in human health. There are more than 1,000 species of microorganisms in the human intestine. The majority of these are anaerobic bacteria, such as clostridium, lactobacillus, *Escherichia coli* and bifidobacterium, and the total number has been estimated as more than 10^14^—ten times higher than the number of human cells [5,6]. Under normal circumstances, these maintain a balanced state and interact with the host, with profound effects on the host’s ability to fight against infections. Previous studies have demonstrated that following damage to the liver there is reduced blood flow through the gut-liver axis, altered bile secretion, and increased epithelial permeability, leading to disruption of both the mucosal barrier and the ecological balance of the gut microflora [7-10]. Previous studies have also found that lactobacillus is significantly reduced, and enterobacter significantly increased [11].

Probiotics are non-pathogenic beneficial flora that act to regulate and maintain a stable intestinal environment and promote micro-ecological balance [12]. VSL#3 is a probiotic mixture which has been frequently referred to in the literature, and contains live lyophilized *Bifidobacterium breve, Bifidobacterium longum, Bifidobacterium infantis, Lactobacillus acidophilus, Lactobacillus plantarum, Lactobacillus paracasei, Lactobacillus bulgaricus* and *Streptococcus thermophilus*.

In this study, we investigated the protective effect of VSL#3 in alcoholic intestinal injury using an animal model. We found that VSL#3 treatment can reduce colonic paracellular permeability and increase the expression of tight junction proteins (ZO-1 and occludin). Furthermore, the intestinal barrier prevents endotoxin and other bacterial products passing from the gut lumen into the portal circulation, and thereby protects against hepatic inflammation.

## Methods

### Rats and treatments

Six- to eight-week-old male WT rats (either littermates or age-matched, 200 ± 10 g at the start of the experiment) were obtained from the Experimental Animal Center, China Medical University. All rats in the study were used strictly in accordance with the National Institutions of Health Guide for the Care and Use of Laboratory Animals. This research was approved by the China Medical University Animals Committee (Approval Number: 2011–1538). They were divided into seven groups, and eight rats in each group.

Rats were deprived of food for 12 hours before induction of acute alcoholic liver injury. For the experiments, rats were divided into seven groups.

In the control group, eight rats were treated twice daily with corn starch which was dissolved in 200 μl of PBS and administered via a gastric tube.

In the alcohol group, eight rats were given 40% alcohol (5 g/kg body weight) through stomach feeding every 12 hours a total of three times.

In the VSL#3, glutamine, heat-killed VSL#3 and VSL#3 + glutamine groups, rats were respectively given VSL#3 (0.6 g/kg body weight), glutamine (0.3 g/kg body weight), heat-killed VSL#3 (0.6 g/kg body weight) or a combination of VSL#3 and glutamine (VSL#3 0.6 g/kg body weight, glutamine 0.3 g/kg body weight) through stomach feeding 30 minutes prior to administration of alcohol as described above.

In the placebo group, rats were treated with PBS through stomach feeding before induction of alcohol liver injury.

The entire small intestine was collected, formalin fixed, and paraffin-embedded.

### Assessment of small intestinal tight junction proteins by electron microscopy

A standard fixation procedure was used for conventional thin section electron microscopy. The procedure involved incubation with OsO4 alone (1 or 2% in phosphate buffer) at 0°C for 30 min. After fixation, the small intestine was washed extensively in Veronal acetate buffer (90 mm, pH 6.0), stained by incubation at 0°C for 60 min in uranylmagnesium acetate (0.5%) in the same buffer, washed again, dehydrated, and embedded. Thin sections were cut at 60 nm with a diamond knife and stained with uranyl acetate and lead citrate for viewing on a 200 CX transmission electron microscopeat 80 kV. High-magnification pictures (×10,000) were taken to evaluate the ultrastructure of small intestinal tight junctions.

### Measurement of TNF-α, endotoxin in plasma

TNF-α levels in the media were assessed using a rat TNF-α ELISA according to the manufacturer’s instructions. TNF-α concentrations were determined using a standard and values were normalized to total DNA present in the well using a DNA quantification kit.

Endotoxin was measured using the Tachypleus Amebocyte Lysate assay. Add 100 μl of endotoxin test solution, endotoxin standard solution and samples into each hole of the micro plate and placed it tachypleus amebocyte lysate, 37°C for 10 minutes. And then add 100 μl tachypleus amebocyte lysate into each of them, read the board at the wavelength of 340 mm once every 30 seconds.

### Western blot analysis

Using snap-frozen small intestine specimens with histologically intact epithelium, we stripped the mucosa from the underlying submucosal tissue, homogenized and sonicated it, and transferred it into ice-cold lysis buffer with a protease inhibitor cocktail for 60 min. Lysates were centrifuged and the protein content of the supernatant was determined using the BCA protein assay kit. Depending on the antibody used, equivalent protein concentrations of 10–75 μg were loaded in each lane of an SDS–polyacrylamide gel. Electrophoretically separated samples were transferred to an immobilon transfer membrane. Membranes were incubated with the respective primary antibodies and a corresponding peroxidase-conjugated secondary antibody. Blots were visualized by chemiluminescence using immobilon Western Chemiluminescent HRP substrate. After detection of specific tight junctions, all membranes were stripped with Restore Western Blot Stripping Buffer, and an immunoblot for β-actin was performed to ensure equal protein loading in each lane. Densitometry was performed for each protein detected in each group.

### RNA isolation and reverse transcription–polymerase chain reaction

All of the plasmids were cloned using PCR. RNA extraction, reverse transcription polymerase chain reaction (RT-PCR), microarray. Primer sequences used in the RT-PCR analyses are presented in supporting information. The following primer pairs were used for amplification:

occludin (sense, 5'-GCTATGAAACCGACTACACGACA-3'; antisense, 5'-ACTCTCCAGCAACCAGCATCT-3').

ZO-1 (sense, 5'-AGGCTATTTCCAGCGTTTTGA-3'; antisense, 5'-AATCCTGGTGGTGGTACTTGC-3').

### Statistical analysis

All data are expressed as mean ± SD and were analyzed using one-way analysis of variance. P <0.05 was considered statistically significant.

## Results and discussion

### Results

#### ***Evaluation of tight junctions using electron microscopy***

**Figure 1:**
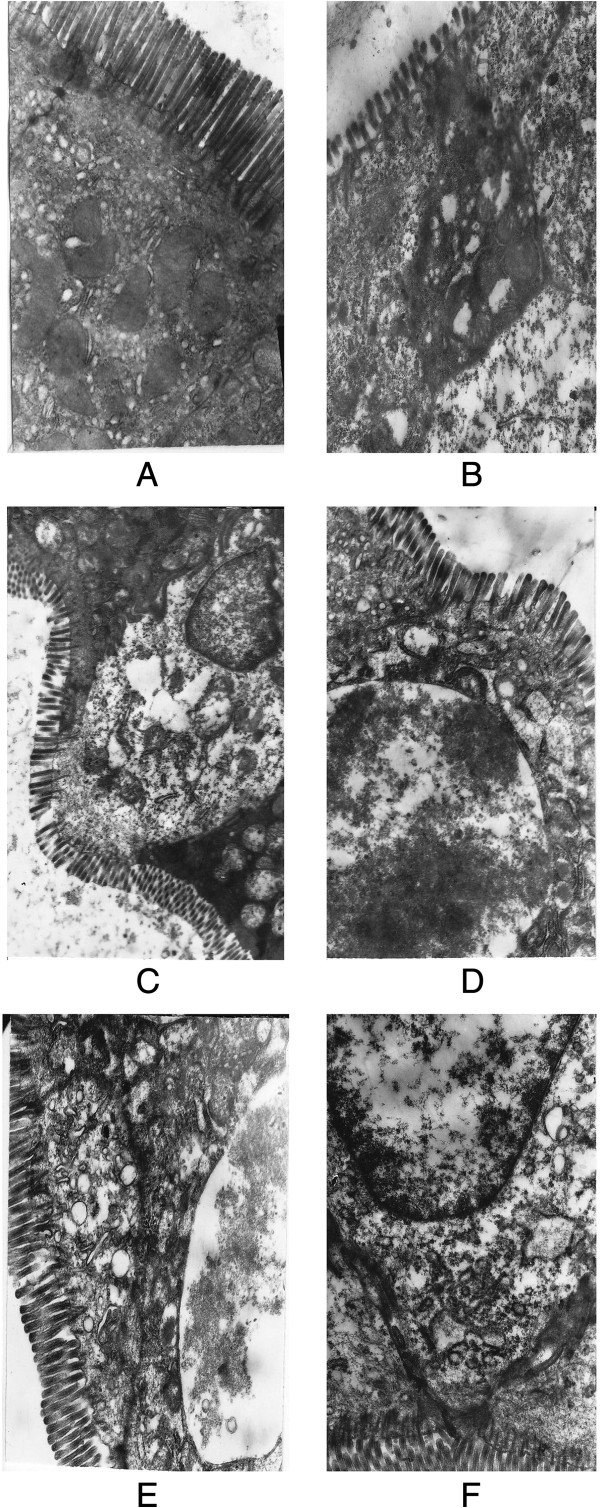
**The expression of tight junctions and microvilli of small intestine cell under electron microscopy. (A)** The expression of tight junctions and microvili in control group; **(B)** The expression of tight junctions and microvili in alcohol group; **(C)** The expression of tight junctions and microvili in glutamine group; **(D)** The expression of tight junctions and microvili in VSL#3 group; **(E)** The expression of tight junctions and microvili in VSL#3+glutamine group; **(F)** The expression of tight junctions and microvili in placebo group.

We studied tight junctions in the small intestine using electron microscopy to establish an index of loss of intestinal barrier integrity. Acute alcohol administration significantly disrupted the architecture of tight junctions of the small intestine. Supplementation with VSL#3, glutamine or heat-killed VSL#3 significantly protected the cyto-architecture of the intestinal barrier, and VSL#3 + Glutamine showed a more significant protective effect on tight junctions than the other treatment groups (Figure 1).

#### ***Endotoxin and TNFα in plasma***

**Figure 2:**
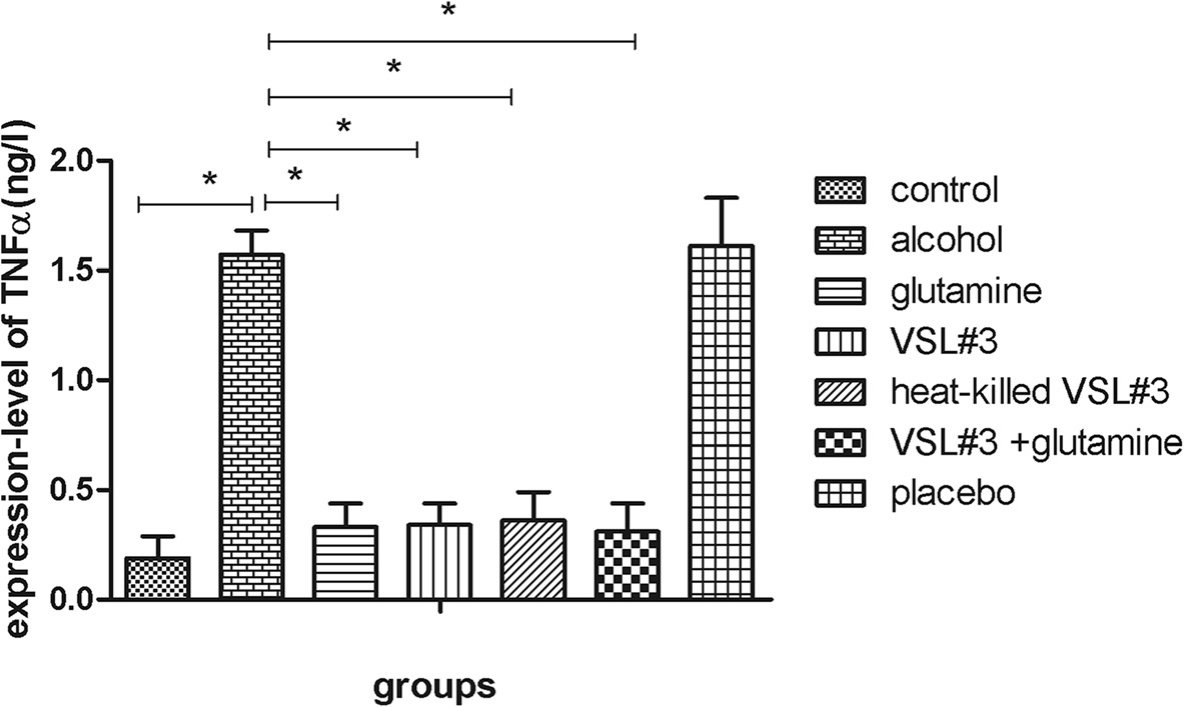
**The expression of TNFα in each group measured by ELISA.** *P < 0.05 versus alcohol group.

We assessed the expression of TNFα in plasma in the seven experimental groups using ELISA. Compared with the control group (174.69 ± 20.68), the expression of TNFα was higher in the alcohol group (383.08 ± 20.21). In the glutamine (211.01 ± 25.87), VSL#3 (201.54 ± 26.56), and heat-killed VSL#3 (197.85 ± 17.97) groups the expression of TNFα was significantly lower than in the alcohol group, and there was no significant difference between these groups. The expression of TNFα in the VSL#3 + glutamine group (195.82 ± 17.19) was lower than in the other three treatment groups, but this difference was not significant (Figure 2).

**Figure 3:**
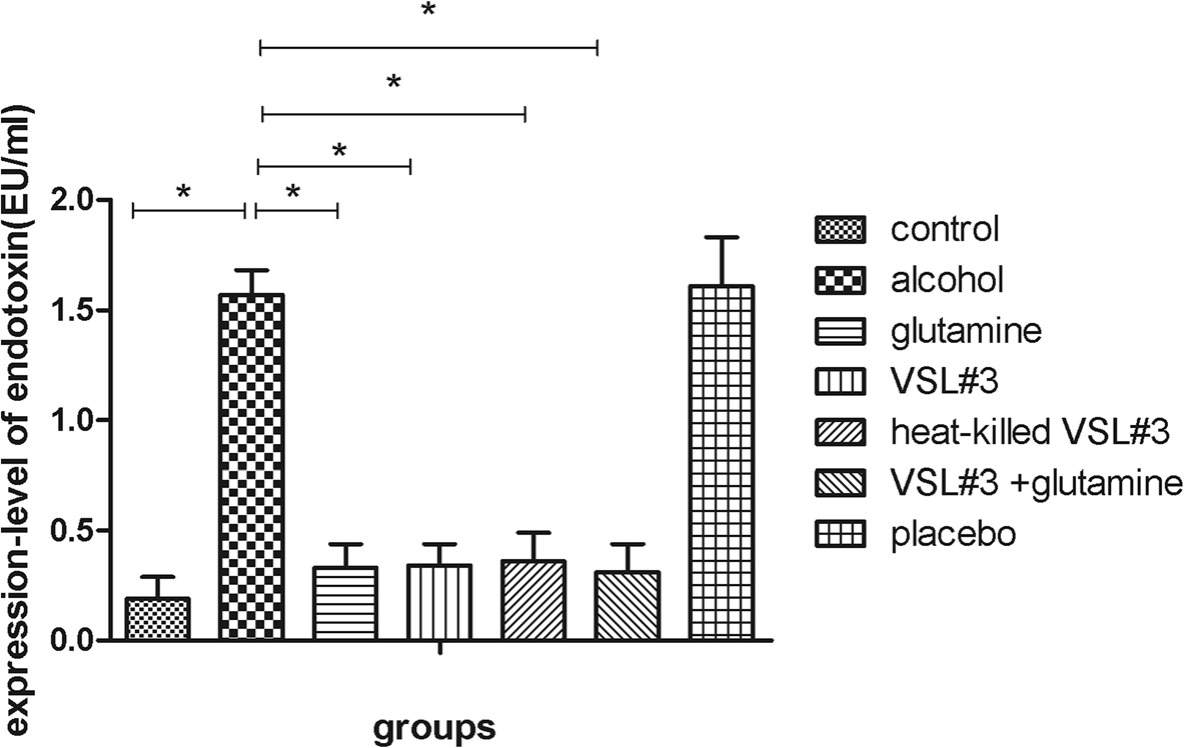
**Levels of endotoxin in each group measured using the Tachypleus Amebocye Lysate assay.** *P < 0.05 versus alcohol group.

We assessed the level of plasma endotoxin in the seven experimental groups using the Tachypleus Amebocyte Lysate assay. Plasma endotoxin was higher in the alcohol group (1.57 ± 0.11) compared with the control group (0.19 ± 0.10). In the glutamine (0.33 ± 0.11), VSL#3 (0.34 ± 0.10), and heat-killed VSL#3 (0.36 ± 0.13) groups, plasma endotoxin was significantly lower than in the alcohol group, and there was no significant difference between these groups. The level of plasma endotoxin in the VSL#3 + glutamine group (0.31 ± 0.13) was lower than the other three treatment groups, but this was not significant (Figure 3).

#### ***Tight junction mRNA and protein expression***

Tight junction mRNA and protein expression was analyzed in the small intestine of the seven groups by RT-PCR and western blotting respectively. The trend of tight junction protein expression was the opposite of the change in endotoxin and TNFα. Specifically, in the alcohol group (occludin mRNA: 0.19 ± 0.04, occludin protein: 0.34 ± 0.06; ZO-1 mRNA 0.19 ± 0.05, ZO-1 protein: 0.19 ± 0.03), expression of mRNA and protein for both occludin and ZO-1 was dramatically lower than the control group (occludin mRNA: 0.56 ± 0.11, occludin protein: 0.79 ± 0.08; ZO-1 mRNA 0.95 ± 0.10, ZO-1 protein: 0.48 ± 0.04).

**Figure 4:**
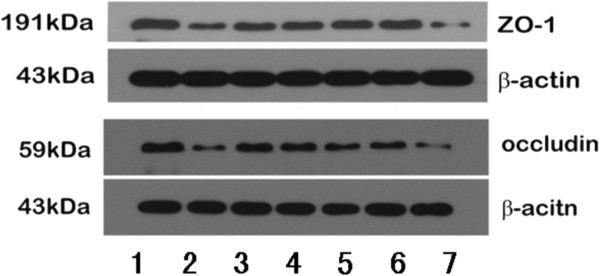
**Western blots of tight junction proteins (occludin and ZO-1).** 1. control group; 2. alcohol group; 3. Glutamine group; 4. VSL#3 group; 5. VSL#3 + Glutamine group; 6. heat-killed VSL#3 group; 7. placebo group.

**Figure 5:**
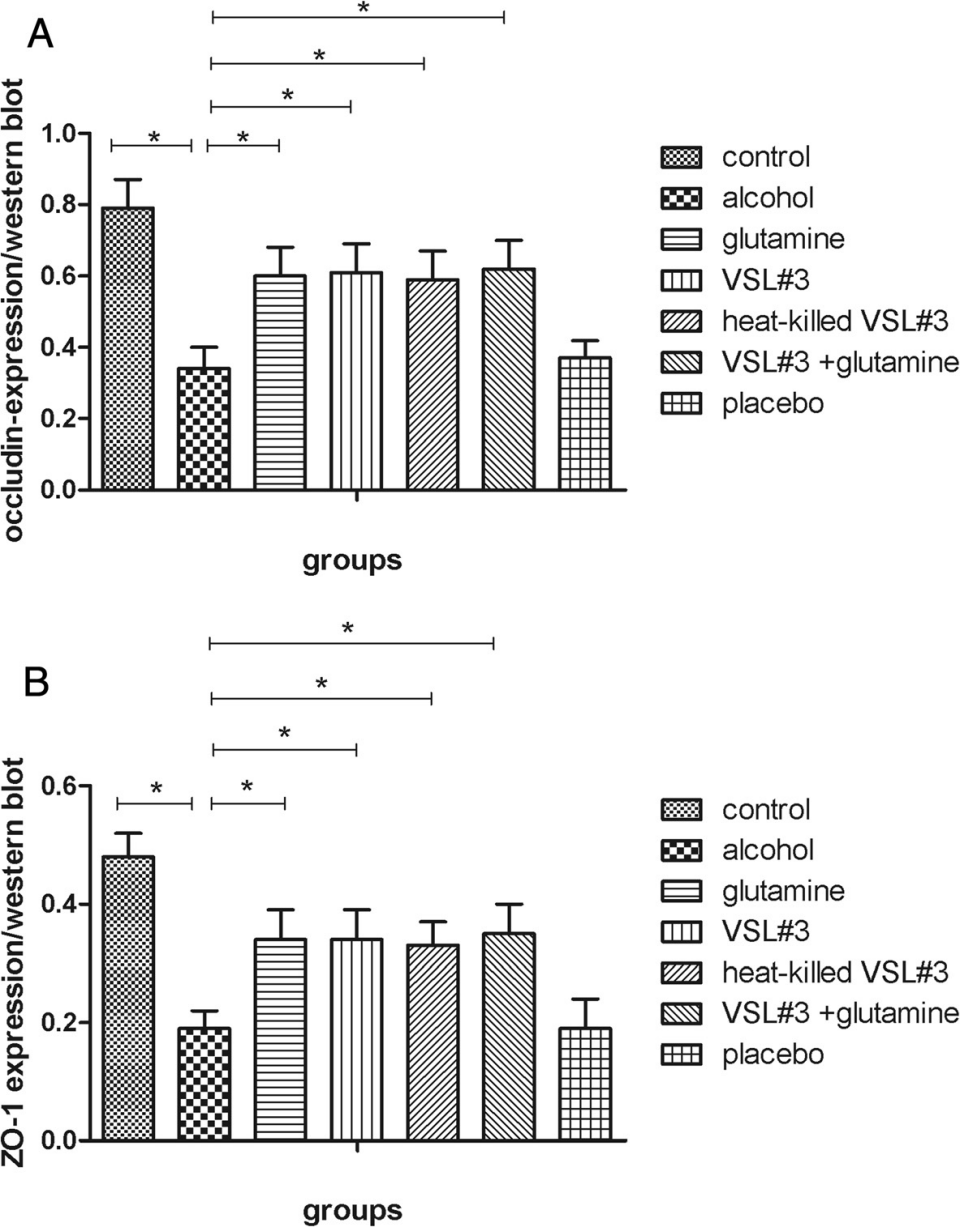
**Western blot densitometry of tight junction protein (occludin and ZO-1) levels in each group. (A)** Western blot densitometry of occludin; **(B)** Western blot densitometry of ZO-1.

**Figure 6:**
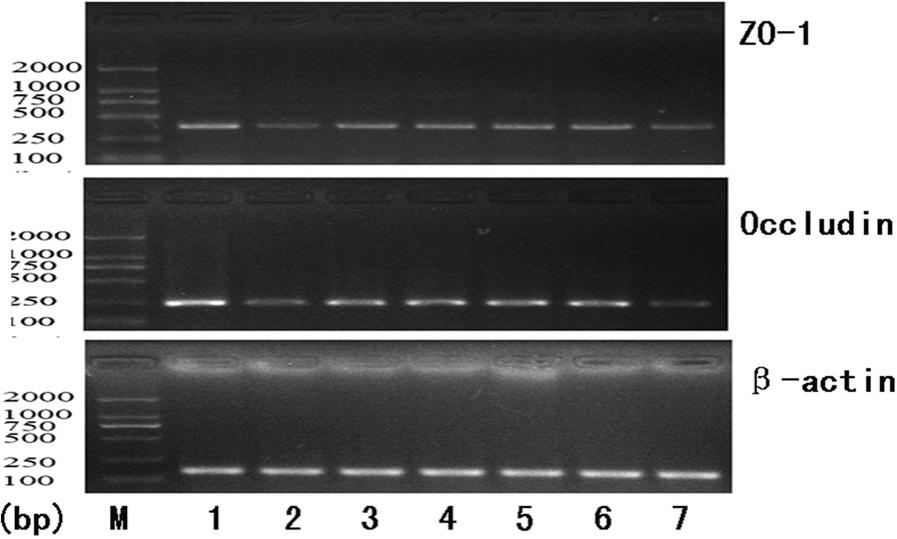
**RT-PCR to measure expression of mRNAs encoding tight junction proteins (occludin and ZO-1).** 1. control group; 2.alcohol group; 3. Glutamine group; 4. VSL#3 group; 5. VSL#3 + Glutaminegroup; 6. heat-killed VSL#3 group ; 7. placebo group.

**Figure 7:**
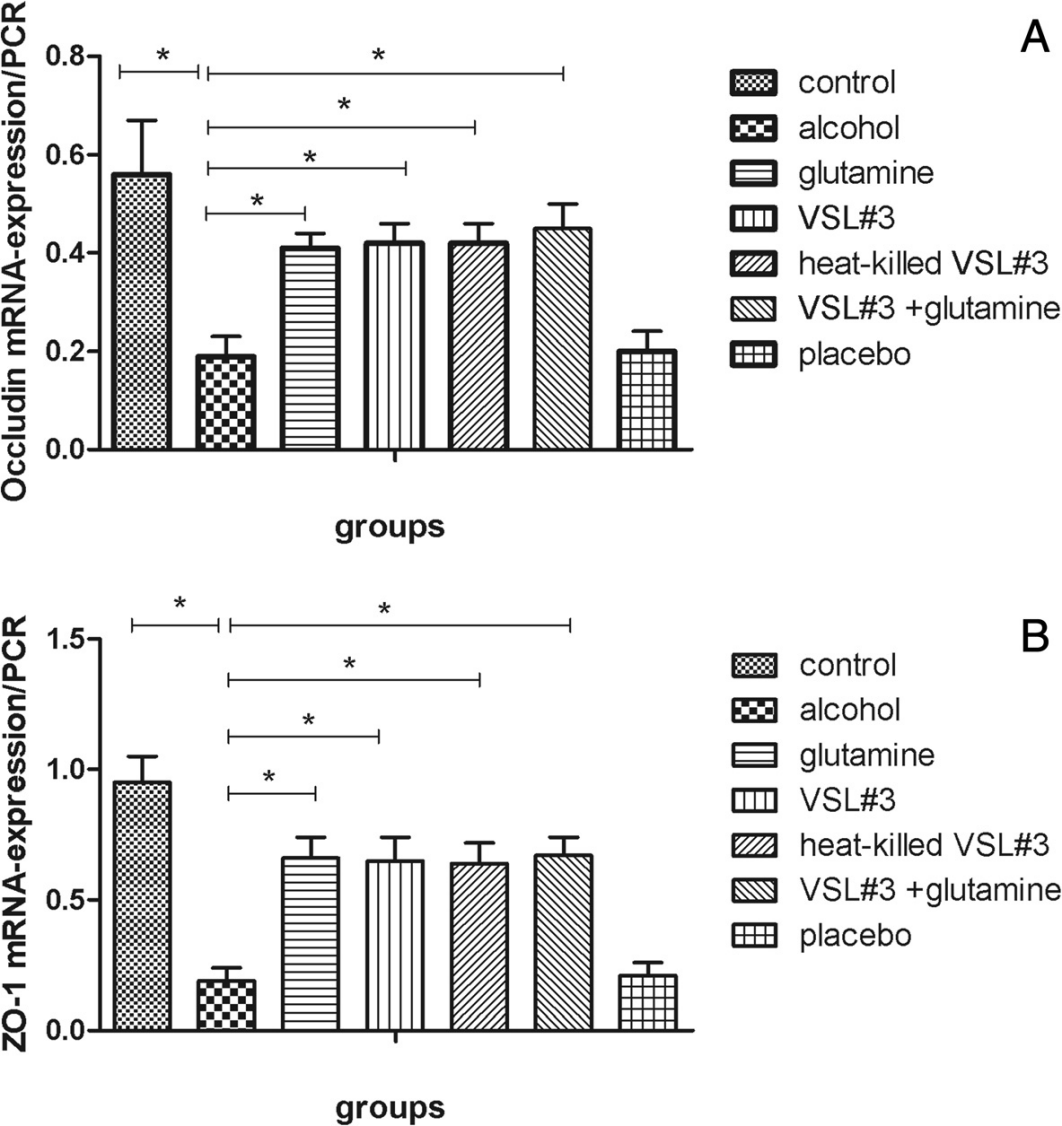
**PCR densitometry of mRNA encoding tight junction proteins (occludin and ZO-1) in each group. (A)** RT-PCR densitometry of occludin mRNA encoding; **(B)** RT-PCR densitometry of ZO-1 mRNA encoding.

In the glutamine (occludin mRNA: 0.41 ± 0.03, occludin protein: 0.60 ± 0.08; ZO-1 mRNA 0.66 ± 0.08, ZO-1 protein: 0.34 ± 0.05), VSL#3 (occludin mRNA: 0.42 ± 0.04, occludin: 0.61 ± 0.08; ZO-1 mRNA: 0.65 ± 0.09, ZO-1: 0.34 ± 0.05), and heat-killed VSL#3 (occludin mRNA: 0.42 ± 0.04, occludin protein: 0.59 ± 0.08; ZO-1 mRNA: 0.64 ± 0.08, ZO-1: 0.33 ± 0.04) groups the expression of tight junction proteins was significantly higher than in alcohol group, and there was no significant difference between these groups. The expression of tight junction proteins in the VSL#3 + glutamine group (occludin mRNA: 0.45 ± 0.05, occludin protein: 0.62 ± 0.08; ZO-1 mRNA: 0.67 ± 0.07, ZO-1 protein: 0.35 ± 0.05) was higher than the other three treatment groups, but this was not significant (Figures 4, 5, 6 and 7).

### Discussion

Recently, the role of the gut microbiota in maintaining human health has attracted increasing attention. However, the relationship between gut microbiota and liver disease has not been extensively investigated. The growing understanding of the functional role of human gut microbiota is showing that this enormous microbial population is instrumental in the control of host energy [13-17]. The human gut microbiota is a complex bacterial community that is relatively stable over time [18-20]. Disruption of the microbiota can increase the risk of several health complications, including loss of colonization resistance against bacterial pathogens [21] and predisposition to autoimmune and allergic diseases [22]. Because the gut is a reservoir for microorganisms and contains more than 1,000 bacterial species, bacterial translocation from the gut due to dysbiosis of the gut microbiota and gut barrier failure may contribute to these infections [23,24]. Members of the Enterobacteriaceae family, *Enterococcus* spp. and the Bacteroides-Prevotella group are potentially pathogenic bacteria, while *Lactobacillus*, *Bifidobacterium* and *F. prausnitzii* are considered beneficial bacterial species for human well-being. Dysbiosis of the gut microbial ecosystem might be associated with the development of endotoxemia and eventually contribute to infections of liver, and TNFα is one of the most important mediators of inflammation.

Our previous study has shown that low grade intestinal inflammation induced by administering wild-type (WT) rats with alcohol results in liver injury. Impairment of the intestinal barrier function is associated with loss of tight junction proteins, including occludin and ZO-1. Tight junctions are the major determinants of paracellular permeability. Disruption of the intestinal barrier would allow endotoxin and other bacterial products in the gut lumen to pass into the portal circulation and thus potentially cause hepatic inflammation and the development of alcoholic steatohepatitis (ASH). This in turn would lead to alcoholic cirrhosis and liver failure, which is a causal factor in the development of alcoholic endotoxemia and hepatitis.

Considering all of these possibilities, in the present study we used a rat model to investigate the effects of VSL#3 administration, to explore its mechanism in the pathogenesis of acute alcohol liver disease, and to compare the effects of VSL#3 with those of glutamine, a combination of VSL#3 + glutamine, and heat-killed VSL#3.

#### ***The therapeutic mechanism of VSL#3 in acute alcohol intestinal disease***

The present study was designed to investigate whether VSL#3 could prevent liver injury by decreasing epithelial permeability. To achieve this we employed a model in which WT rats were fed with VSL#3 before administration of alcohol. Our results demonstrate that expression of the tight junction proteins ZO-1 and occludin was decreased in acute alcohol liver disease, and VSL#3 treatment suppressed this effect by regulating the ecological balance of the gut microflora, preventing endotoxin and other bacterial products in the gut lumen from passing into the portal circulation and down-regulating the expression of TNFα, which could otherwise down-regulate the expression of tight junction proteins and increase epithelial permeability, then endotoxin and other bacterial products pass from the gut lumen into the portal circulation, and lead to hepatic inflammation. Our results therefore suggest that probiotic-induced protection of epithelial barrier function is through prevention of changes in tight junction protein expression.

#### ***Assessing the effect of VSL#3, glutamine, VSL#3 + glutamine and heat-killed VSL#3***

Glutamine is a conditionally essential amino acid with immunomodulatory properties. It has a protective role in intestinal injury models, and may regulate proliferation of intestinal epithelial cells by modulating responsiveness to growth factors [25,26]. Small intestinal mucosa becomes atrophic when the gut is deprived of glutamine, for example during total parenteral nutrition [27]. Glutamine depletion can increase permeability of the gut which promotes translocation of luminal bacteria and toxins [28]. Glutamine has been shown to protect intestinal epithelial cells during physiological stress because it is required for stress-induced heat shock protein expression [29,30] such as in experimental enterocolitis [31,32]. Glutamine most likely protects the gut via mucosal healing and a decrease in bacterial translocation [33]. Moreover, some studies have reported that glutamine down-regulated the intestinal inflammatory response in experimental models [31,34-36] by modulating the nuclear factor-κB (NF-κB) pathway [37-40].

The aim of the study was therefore to assess the effects of glutamine and VSL#3, either alone or in combination, on acute alcohol liver disease, and to compare the effects of VSL#3 and heat-killed VSL#3. We found that both VSL#3 and heat-killed VSL#3 were as effective as glutamine in the treatment of acute alcohol liver disease, whereas the combination of VSL#3 and glutamine therapy efficacy was more effective than either agent alone (although it showed no significant difference compared with the other groups).

## Conclusions

The probiotic mixture VSL#3 and heat-killed VSL#3 were as effective as the traditional agent glutamine in rats with experimental acute alcohol liver disease, whereas the combination of VSL#3 and glutamine was more effective than either alone. All of these treatments can prevent endotoxin and other bacterial products in the gut lumen from passing into the portal circulation, decrease the production of TNFα and increase the expression of tight junction proteins, thus reducing paracellular intestinal permeability.

## Competing interest

The authors declare that they have no competing interests.

## Authors’ contributions

BC and LS conceived and designed the study. BC and YW did the experiment technology. BC, JT, and BW did statistical analyses and interpreted results. All drafts of the reports, including the final version, were written by BC and LS and revised by BW. All authors read and approved the final paper.

## Pre-publication history

The pre-publication history for this paper can be accessed here:

http://www.biomedcentral.com/1471-230X/13/151/prepub
